# Optimized Silica-Binding Peptide-Mediated Delivery of Bactericidal Lysin Efficiently Prevents *Staphylococcus aureus* from Adhering to Device Surfaces

**DOI:** 10.3390/ijms222212544

**Published:** 2021-11-21

**Authors:** Wan Yang, Vijay Singh Gondil, Dehua Luo, Jin He, Hongping Wei, Hang Yang

**Affiliations:** 1 State Key Laboratory of Agricultural Microbiology, College of Life Science and Technology, Huazhong Agricultural University, Wuhan 430070, China; yw13618649009@hotmail.com (W.Y.); luode_hua@webmail.hzau.edu.cn (D.L.); hejin@mail.hzau.edu.cn (J.H.); 2 CAS Key Laboratory of Special Pathogens and Biosafety, Center for Biosafety Mega-Science, Wuhan Institute of Virology, Chinese Academy of Sciences, Wuhan 430071, China; vjgondal@gmail.com (V.S.G.); hpwei@wh.iov.cn (H.W.); 3 University of Chinese Academy of Sciences, Beijing 100049, China

**Keywords:** lysin, *Staphylococcus aureus*, silica-binding peptide, antimicrobial agents immobilization, surface functionalization, antimicrobial agents, biofilm

## Abstract

Staphylococcal-associated device-related infections (DRIs) represent a significant clinical challenge causing major medical and economic sequelae. Bacterial colonization, proliferation, and biofilm formation after adherence to surfaces of the indwelling device are probably the primary cause of DRIs. To address this issue, we incorporated constructs of silica-binding peptide (SiBP) with ClyF, an anti-staphylococcal lysin, into functionalized coatings to impart bactericidal activity against planktonic and sessile *Staphylococcus aureus*. An optimized construct, SiBP1-ClyF, exhibited improved thermostability and staphylolytic activity compared to its parental lysin ClyF. SiBP1-ClyF-functionalized coatings were efficient in killing MRSA strain N315 (>99.999% within 1 h) and preventing the growth of static and dynamic *S. aureus* biofilms on various surfaces, including siliconized glass, silicone-coated latex catheter, and silicone catheter. Additionally, SiBP1-ClyF-immobilized surfaces supported normal attachment and growth of mammalian cells. Although the recycling potential and long-term stability of lysin-immobilized surfaces are still affected by the fragility of biological protein molecules, the present study provides a generic strategy for efficient delivery of bactericidal lysin to solid surfaces, which serves as a new approach to prevent the growth of antibiotic-resistant microorganisms on surfaces in hospital settings and could be adapted for other target pathogens as well.

## 1. Introduction

Medical devices play a pivotal role in the management of healthcare-associated patients in modern medicine. Indwelling urinary and central venous catheters are the most frequently employed invasive devices, which have revolutionized medical treatment, especially post-operative care [1]. These catheters remain continuously interacting with body fluids for a long duration, which is critical for the success of these implants [2]. However, long-term catheterization also favors microbial colonization and dissemination, leading to the failure of implant and further increasing the treatment cost and the severity of ailment [3]. For instance, a 3–6% per day increased risk of bacterial colonization has been estimated in urinary catheterization and 7–10 days of catheterization may result in catheter-associated infections in 50% of the hospitalized patients [4,5]. Bacterial colonization on surface of central venous catheters presents more serious complications as compared to urinary catheters, as relatively small number of colonized bacteria can successfully establish the central line associated blood stream infections [6], which commonly links to higher life-threatening risk and cost [7]. 

In general, different types of implantable biomedical device-related infections (DRIs) have linked to varied bacterial species. For example, cardiovascular implantable devices and joint prosthesis are often colonized by Staphylococci, Streptococci, Enterococci, and *Candida* spp. [8]. However, Staphylococci are one of the major causes of medical DRIs due to strong capacity to form device-related biofilms, and the most pathogenic and aggressive one is methicillin-resistant *S. aureus* (MRSA) [9]. Various strategies have been employed to reduce bacterial colonization on surfaces of devices, such as increased hygiene, optimized dwelling measures, and biocidal agent-based surface modifications [10]. However, the antimicrobial resistance and biofilm-forming ability of *S. aureus* brought the efficacy of these strategies into question [11]. Therefore, designing novel antibacterial and antibiofouling coating is needed to counter the mounting menace of drug-resistant *S. aureus*-mediated DRIs. 

It has been demonstrated in the past two decades that the bacteriophage-encoded peptidoglycan hydrolase, i.e., endolysin or lysin, is a promising candidate to overcome antimicrobial resistance [12,13]. The primary function of lysin is to release the virion progeny from the host cytoplasm by digesting its peptidoglycan, but in recent years, lysin is immensely exploited as an antibacterial agent due to its unique mechanisms of action. A good example is the chimeric lysin ClyF that possesses high activity against planktonic and biofilm *S. aureus* both in vitro and in vivo models [14]. ClyF composes the catalytic domain (CD) from Ply187 lysin (GenBank: CAA69022.1, UniProtKB: O56785, the N-terminal 157 aa) with a putative CHAP (cysteine, histidine-dependent amidohydrolase/peptidase) catalytic activity and the cell wall binding domain (CBD) from PlySs2 lysin (NCBI No.: WP_170238997.1, UniProtKB: A0A0Z8Y0I3, the C-terminal 99 aa) that possesses high affinity for *S. aureus*. Because of rapid activity, high specificity, proteinaceous nature, and not yet reported case of resistance [15], lysin could be presented as a potent alternative agent for immobilization on surface of devices to prevent DRIs. 

Lysins have been well investigated in various in vitro and in vivo infection models against a range of potent Gram-positive and Gram-negative pathogens over the decades [16,17,18,19,20,21]. Continuous work has well established that the second generation of lysin could be engineered to possess improved bactericidal activity and/or broad spectrum of function [22,23]. Whilst the third generation of lysin could be continuously engineered and developed to gain improved pharmacological properties [24]. Few studies demonstrated the activity of phage-derived lysins stored in complexes with block-copolymers of poly-L-glutamic acid and polyethylene glycol [25], or after fusing with matrix-binding protein, such as cellulose binding domain [26]; however, there is a knowledge void in the activity as well as the possibility of immobilized lysins on solid surfaces. The current study was designed to fill the existing knowledge void and investigated the potential of bactericidal lysins immobilization on biomedical surfaces as a means to combat medical DRIs.

**Figure 1:**
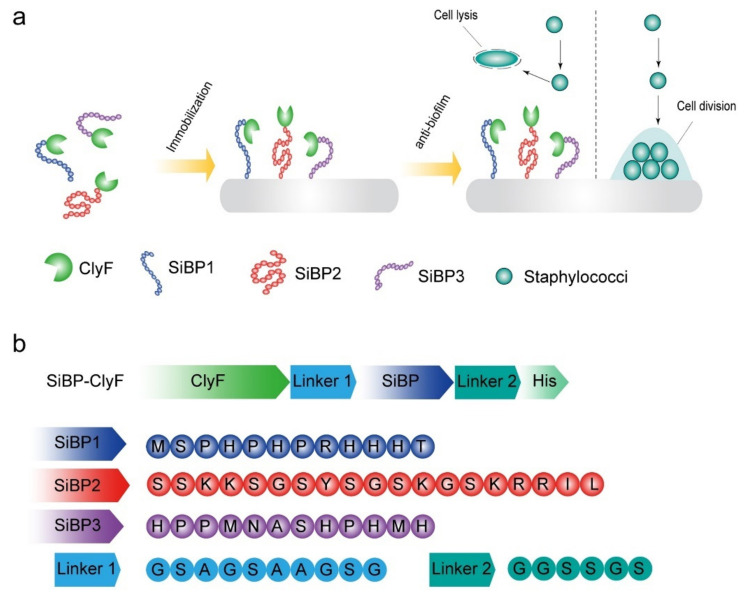
Schematic diagram of experimental design. (**a**) Silica-binding peptide-mediated fixation of ClyF imparts antistaphylococcal propriety to solid surfaces. (**b**) Construction and composition of SiBP-fused ClyF variants. The amino acid sequences of SiBP1, SiBP2, SiBP3, linker between ClyF and SiBP (Linker 1), and linker between SiBP and His tag (Linker 2) are shown.

Short solid binding peptides are popular molecular linkers for the fabrication of bioactive proteins onto solid surfaces via multiple weak noncovalent interactions [27]. These peptides increase the affinity of bioactive proteins for their targeted solid matrix and confers orientation, flexibility, and directionality to proteins without impeding their activity [28,29]. Therefore, in the present study, ClyF was used as a model lysin to be modified with three different silica-binding peptides (SiBPs) [14] to convene the conception of bactericidal lysin-functionalized surfaces as a new approach to block device-related bacterial colonization (Figure 1). Specifically, three peptides reported to have high affinity for silica surfaces were selected fusing to the C-terminal of ClyF, and SiBP-fused ClyF variants were then immobilized on various silicone-containing surfaces and evaluated for their antibacterial and antibiofilm activities under static and dynamic conditions.

## 2. Results

### 2.1. Construction and Biochemical Characterization of ClyF Variants

Previously, we demonstrated that the chimeric lysin ClyF is highly active against various staphylococcal strains [14], highlighting its potential as an alternative antistaphylococcal agent. Considering the epidemiology and threats of staphylococci-primed device-related infections (DRIs) in model healthcare settings, we further explored the antistaphylococcal capacity of surface-immobilized ClyF by using solid surface directed silica-binding peptide (SiBP). To this end, three peptides varying in charge and length, i.e., a 12-residue peptide SiBP1 (MSPHPHPRHHHT; Z = +1) [30], a 19-residue peptide SiBP2 (SSKKSGSYSGSKGSKRRIL; Z = +6) [31], and another 12-residue peptide SiBP3 (HPPMNASHPHMH; Z = 0) [32], were selected fusing to the C-terminal of ClyF lysin (Figure 1). To adjust the compatibility of ClyF lysin and silica-binding peptide, a separate flexible linker was incorporated before and after each peptide sequence, and a C-terminal 6×his-tag was attached for affinity purification of each construct (Figure 1). All ClyF constructs were expressed in *E. coli* BL21(DE3) cells as soluble proteins and purified using Ni-NTA affinity chromatography (Figure S1).

**Figure 2:**
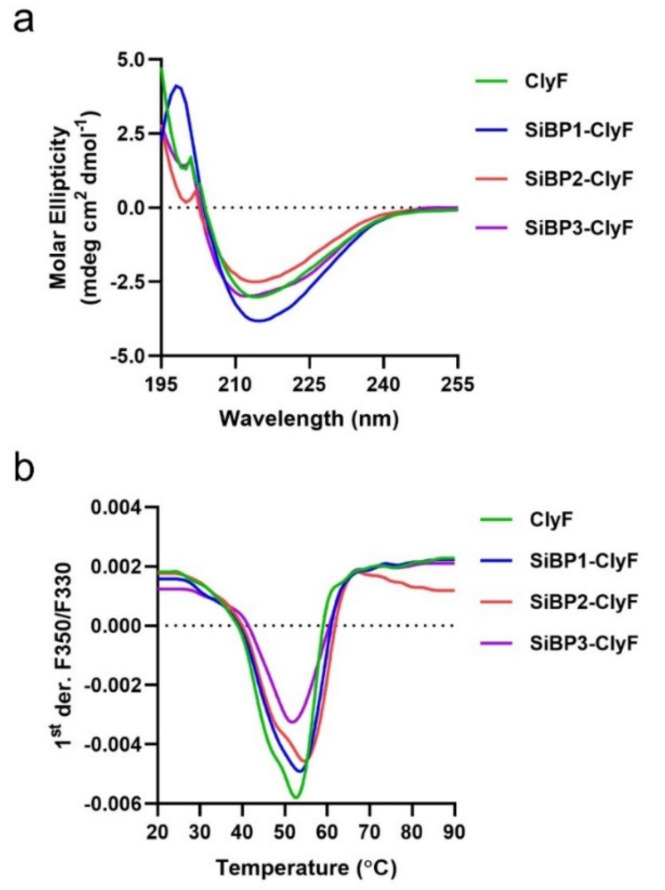
Biochemical properties of ClyF and its variants. (**a**) Circular dichroism spectra of ClyF and its variants. The UV spectra of ClyF and its variants were scanned from 190–260 nm (0.1 cm path length) at room temperature. (**b**) Thermal unfolding profiles for ClyF and its variants. The profiles of all proteins were determined by nanoDSF from 25–90 °C. The *Y*-axis represents the first derivative of the ratio of fluorescence at 350 nm and 330 nm.

Because the bactericidal activity of ClyF depends on the proper folding of its two functional domains, we thus assessed the effects of SiBP on the structural conformation of ClyF variants by circular dichroism. Results showed that the spectra of ClyF, SiBP2-ClyF and SiBP3-ClyF almost resemble each other and that the spectra of SiBP1-ClyF showed minor differences due to difference in the molar ellipticity intensities (Figure 2a). However, all four proteins had UV peaks at 215 nm, suggesting similar folding in all four. Additional analysis revealed that the composition of the secondary structural components for all four enzymes is nearly identical (Table S1), indicating that SiBP-fusing only introduces a minor influence on the folding profile of ClyF domains. We further used RoseTTAFold to perform structural predictions for ClyF and its variants. Each prediction showed a similar structure with a hydrophobic cleft in the catalytic domain and an Ig-like structure in the CBD (Figure S2). However, the predicted surface shape of SiBP2-ClyF is a bit different from that of the other variants, showing a L-like shape, while the others displayed a V-like shape (Figure S2).

Next, we evaluated the influence of these peptides on the thermostability of ClyF using nano-differential scanning fluorimetry. Results showed that the thermal transition temperature increases from 52.1 °C for ClyF to 54.3 °C for SiBP2-ClyF, the variant with the highest positively charged peptide, while the other two ClyF variants, SiBP1-ClyF and SiBP2-ClyF, exhibit similar thermal transition temperatures with their parental ClyF lysin (Figure 2b and Table S2). The single peaks noted for ClyF and its three SiBP-fused variants indicate that the two constitutive domains of ClyF unfold together in all four (Figure 2b). 

### 2.2. Bactericidal Activities of Free ClyF Variants

**Figure 3:**
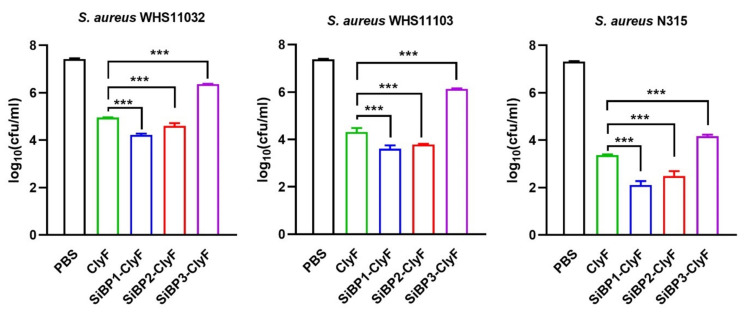
Bactericidal activities of ClyF and its SiBP-fused variants. *S. aureus* strains WHS11032, WHS11103, and N315 were treated with equal molar concentration (0.7 μM) of each protein for 1 h at 37 °C, residual number of viable bacteria was determined by plating serial dilutions on LB agar. Data are shown as means ± standard deviations and *** represents *p* < 0.001.

Initially, we tested the bacteriolytic of ClyF and its variants against eight representative *S. aureus* strains with different genetic profiles (Table S3), results showed that SiBP1-ClyF and SiBP2-ClyF exhibit improved lytic activities in all strains tested compared to the native ClyF (Figure S3). In contrast, an impaired activity was observed in SiBP3-ClyF, which could be associated to its slightly different circular dichroism curve (Figure 2a) and 3D modeling structure (Figure S2). We then evaluated the bactericidal activities of ClyF and its variants against three staphylococcal strains, WHS11032, WHS11103, and N315, under equal molar concentrations. In consistent with the bacteriolytic assays, the variant SiBP3-ClyF maintained impaired bactericidal activity compared to that of the parental ClyF lysin, effecting a 1- to 3-log10 decrement in bacterial viability in 60 min, depending on the strain (Figure 3). In contrast, SiBP1-ClyF and SiBP2-ClyF exhibited significantly (*p* < 0.001) elevated bactericidal activity, effecting a 1.36- and 0.87-log10 increase in killing activity for *S. aureus* N315, respectively, compared to the activity of their parental ClyF (Figure 3). As both SiBP1 (Z = +1) and SiBP2 (Z = +6) are positively charged, presumably, the neutrally charged SiBP3 (Z = 0) prevents the direction of the cell-wall binding domain (CBD) in SiBP3-ClyF to its bacterial cell wall substrates.

### 2.3. Bactericidal Capacities of Immobilized ClyF Variants against Planktonic S. aureus

**Figure 4:**
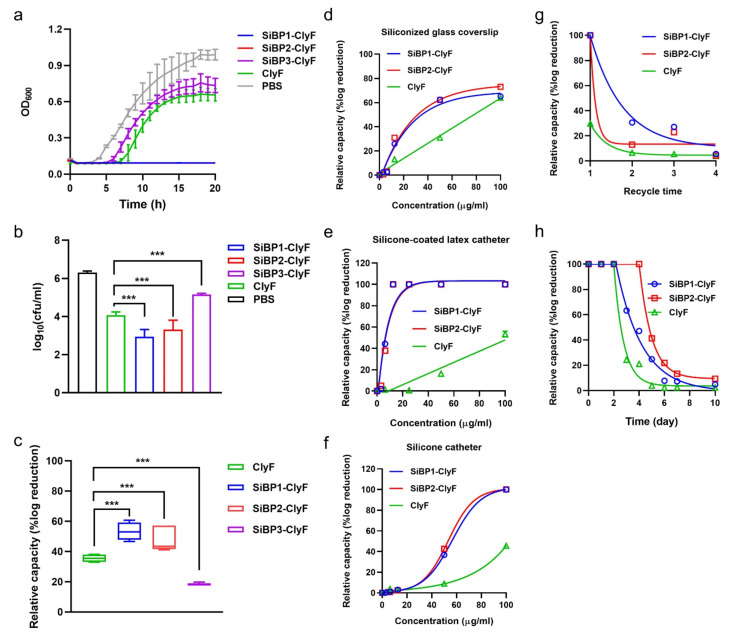
Bactericidal activities of immobilized ClyF and its variants against planktonic *S. aureus* on different surfaces. (**a**) Bacteriostatic activities of ClyF and its variants immobilized on siliconized glass surface against *S. aureus* N315. (**b**,**c**) Bactericidal capacities of ClyF and its variants immobilized on siliconized glass surface against *S. aureus* N315. ClyF and its SiBP-fused variants were immobilized on siliconized glass surfaces and cocultured with *S. aureus* N315 for 1 h at 37 °C, residual number of viable bacteria was determined by plating serial dilutions on LB agar (**b**), and the relative capacity of each protein was presented as the percentage of log reduction in lysin-immobilized groups in comparison to the mock-immobilized PBS-treated controls (**c**). (**d**–**f**) Bactericidal capacities of ClyF and its variants on different immobilized surfaces. Different concentrations of ClyF, SiBP1-ClyF, and SiBP2-ClyF were immobilized on surfaces of siliconized glass coverslip (**d**), silicone-coated latex catheter (**e**), and silicone catheter (**f**) for 2 h at room temperature, the bactericidal capacity of each surface after co-culture with *S. aureus* N315 for 1 h was then determined by plating assay. (**g**) Recycle capacity of ClyF, SiBP1-ClyF, and SiBP2-ClyF immobilized glass 96-well plates. (**h**) Bactericidal activities of ClyF, SiBP1-ClyF, and SiBP2-ClyF immobilized glass 96-well plates after stored different times at 4 °C. Data are shown as means ± standard deviations and *** represents *p* < 0.001.

Next, we compared the bacteriostatic activities of immobilized ClyF and its variants against the growth of planktonic *S. aureus*, under an equal molar concentration. As shown in Figure 4a, immobilized SiBP1-ClyF and SiBP2-ClyF showed significantly improved bacteriostatic activities, with rare notable bacterial growth even after 20 h of co-incubation at 37 °C, as compared to the native ClyF. In contrast, the variant SiBP3-ClyF showed attenuated bacteriostatic ability, priming a 2-h-earlier visible growth of *S. aureus* N315, compared to the parental ClyF (Figure 4a). Notably, compared to the PBS-treated group, minor bacteriostatic activity was observed in ClyF-immobilized wells, implying a non-specific binding of ClyF to supporting surfaces. In addition, we determined the viable bacterial number in lysin-immobilized wells after co-cultured for 1 h to further confirm their bacteriostatic activities. Results showed that, the variants SiBP1-ClyF and SiBP2-ClyF exhibit improved bactericidal activities, leading to a killing of 3.37- and 2.99-log10 (Figure 4b), corresponding to a reduction of 53.36% and 47.39% in log10 (Figure 4c), respectively, compared to the killing of 2.24-log10 and reduction of 35.52% in log10 in the parental ClyF. As expected, SiBP3-ClyF maintained impaired bactericidal activity in comparison to the wild type ClyF (Figure 4b,c), which is relatively consistent with the attenuated bacteriostatic activity in immobilized SiBP3-ClyF.

As the above observations collectively showed that immobilized SiBP1-ClyF and SiBP2-ClyF possess a higher relative capacity on the solid surface than SiBP3-ClyF and ClyF (Figure 4c), we further characterized their performance in multiple clinically relevant surfaces, including siliconized glass, silicone-coated latex catheter, and silicone catheter. Results showed that immobilized SiBP1-ClyF and SiBP2-ClyF exhibit similar dose-dependent anti-staphylococcal responses and significantly elevated capacities in all three surfaces tested, compared to the parental ClyF (Figure 4d–f). Comparable capacities were observed in siliconized glass coverslip under higher immobilization concentration (up to 100 µg/mL) for all three lysins (Figure 4d), indicating strong non-specific binding of positively charged ClyF CBD to glass surfaces. On the surface of silicone-coated catheter, SiBP1-ClyF and SiBP2-ClyF showed high bactericidal activities under low concentrations, almost achieving their maximal bactericidal capacity at 12.5 µg/mL for both proteins, causing a reduction of 5.52-log10 in viable bacterial number (>99.999% removing efficacy), compared to the activity of ClyF (Figure 4e). Likewise, high anti-staphylococcal activities were also observed in SiBP1-ClyF- and SiBP2-ClyF-coated surfaces of silicone catheter (Figure 4f). 

In addition, a recycle evaluation assay showed that the relative capacity of lysin-functionalized surfaces decreased along with the recycle times, and rare bactericidal activity was observed after 4 recycles (Figure 4g). Nonetheless, the bactericidal activity of SiBP1-ClyF-immobilized wells after three recycles was still comparable to that of the ClyF-immobilized wells in its first cycle (Figure 4g). Like other protein-functionalized surfaces, immobilized lysins lose bactericidal activity in a time-dependent manner and were almost inactivated after 7 days of storage at 4 °C (Figure 4h), although SiBP1-ClyF- and SiBP2-ClyF-immobilized wells showed improved tolerance than that of ClyF-coated wells. Taken together, immobilization of ClyF on silicone-containing surfaces via SiBP1 and SiBP2 could bring high antimicrobial activity and stability against planktonic *S. aureus*.

### 2.4. Antibiofilm Capacity of Immobilized SiBP1-ClyF against Static S. aureus Biofilms

**Figure 5:**
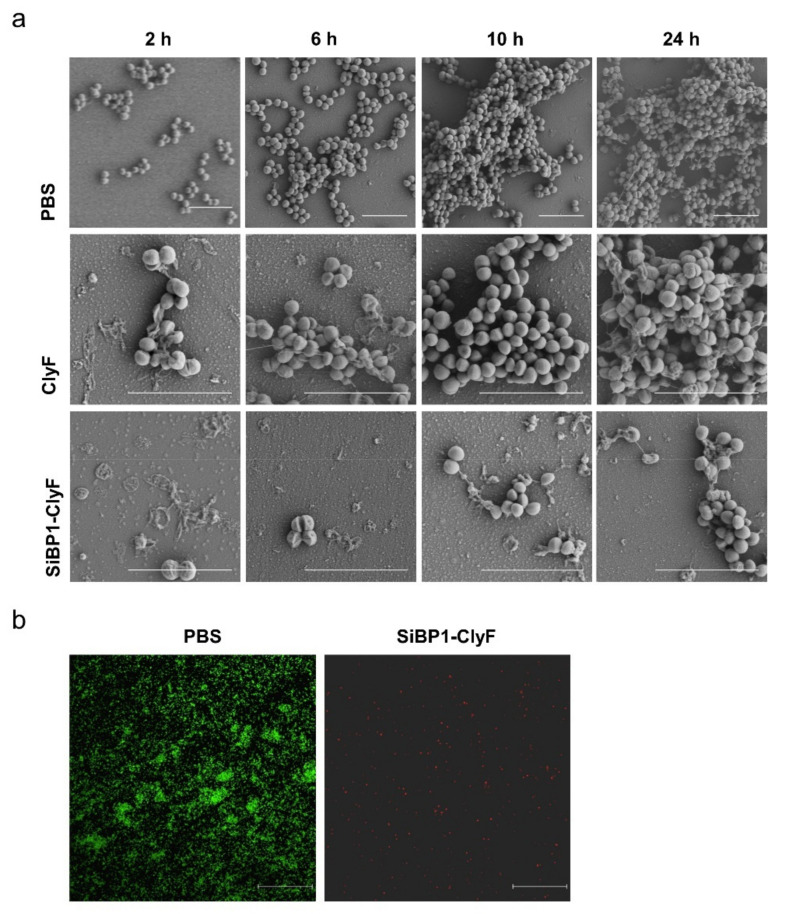
Antibiofouling activity of SiBP1-ClyF-immobilized surfaces against static *S. aureus* biofilms. (**a**,**b**) Analysis of antibiofilm activity of SiBP1-ClyF-immobilized surfaces. Siliconized glass coverslips were coated with 100 µg/mL of SiBP1-ClyF for 2 h at room temperature, and then incubated with *S. aureus* N315 in TBSG at 37 °C. Biofilms developed at 2, 6, 10, and 24 h were analyzed by a JSM-6390 scanning electron microscope (**a**). Biofilms developed at 10 h were further stained with Live/Dead bacterial viability kit and visualized by confocal fluorescence microscope (**b**). Bar scalar: 5 µm.

As *S.* *aureus* biofilm is the most common cause of surgical site infections and medical DRIs initiated by bacterial adherence to surfaces [33], we thus ascertained the antibiofilm activity of immobilized ClyF, harboring the conception that immobilized ClyF could kill surface Staphylococci and thus prevent biofilm formation. Because immobilized SiBP1-ClyF shows the highest bactericidal capacity against *S. aureus* (Figure 4c), the antibiofouling capacity of ClyF-functionalized surface was carried out by using SiBP1-ClyF in our subsequent studies. In this regard, SiBP1-ClyF was immobilized on surface of siliconized glass coverslips and cocultured with *S. aureus* N315 in the biofilm formation medium. Resulted staphylococcal biofilms of different ages (2, 6, 10, and 24 h), corresponding to different developing stages of *S.* *aureus* biofilms [34], were analyzed by SEM. As shown in Figure 5a, mock-immobilized groups evidenced the development of *S. aureus* biofilms from initial attachment, multiplication, and exodus to mature biofilms. In contrast, SiBP1-ClyF immobilized group showed a significantly retard development of *S. aureus* biofilms, with fewer *S. aureus* attached to lysin-functionalized surfaces, visible cell deformation, and dead ghost cells caused by the lysis of SiBP1-ClyF (Figure 5a). Notably, the ClyF-coated group showed reduced *S. aureus* attachment and visible signs of cell lysis, probably due to the non-specific bound of ClyF to glass surface, but rare inhibitory effects on the maturation of *S. aureus* biofilms were observed (Figure 5a). To more accurately reflect the antibiofilm capacity of SiBP1-ClyF-immobilized surfaces, we stained *S. aureus* biofilms aged 10 h by Live/Dead bacterial viability kit and imaged using confocal fluorescence microscopy. Results showed that a lawn of confluent bright green stained cells, indicating viable *S. aureus*, was observed in the mock-immobilized PBS-treated control group (Figure 5b), which could be correlated with the high bacterial load and the successful establishment of bacterial biofilms. In contrast, rare green fluorescent signal was detected in SiBP1-ClyF immobilized group, but multiple scattered red fluorescent spots were observed, representing dead *S. aureus* cells (Figure 5b). These observations collectively showed that the SiBP1-ClyF-immobilized surface has a potent antibiofouling capacity against static *S. aureus* biofilms.

### 2.5. Antibiofilm Capacity of Immobilized SiBP1-ClyF against Dynamic S. aureus Biofilms

**Figure 6:**
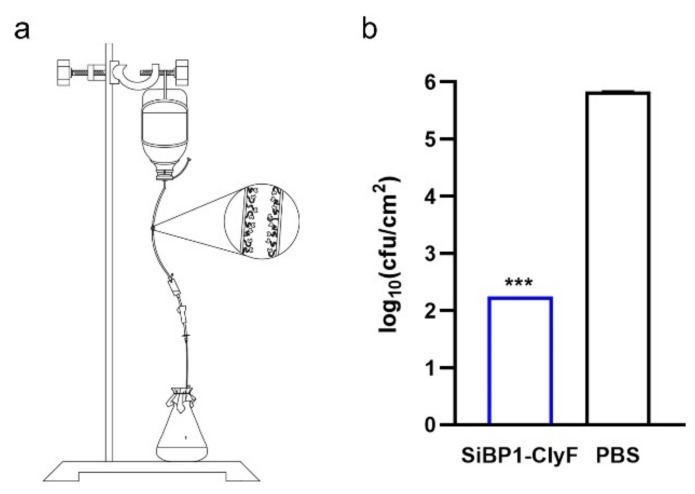
Antibiofouling activity of SiBP1-ClyF-immobilized surfaces against dynamic *S. aureus* biofilms. (**a**) Schematic diagram of fabricated dynamic *S. aureus* biofilm model. Silicone catheter (~5 cm length) was coated with 100 µg/mL of SiBP1-ClyF for 2 h at room temperature, and then incubated with ~10^6^ CFU/mL of *S. aureus* N315 for 3 h at room temperature. Afterwards, the silicone catheter was inserted into a peripherally inserted central catheter and flowed with LB at a fluid speed of 0.6 mL/min for 24 h to allow biofilm development. Finally, silicone catheters were removed and underwent an ultrasound bath for 10 min to disturb established biofilms. Resulted viable bacterial number was analyzed by plating serial dilutions on LB agar. PBS-treated mock-coated silicone catheter was used as control. (**b**) Antibiofilm capacity of immobilized SiBP1-ClyF on surface of silicone catheter. Data are shown as means ± standard deviations and *** represents *p* < 0.001.

To mimic the in vivo antibiofilm capacity of immobilized SiBP1-ClyF on clinically relevant surfaces, we established a dynamic biofilm model using silicone catheter and evaluated the performance of SiBP1-ClyF. To this end, 100 µg/mL SiBP1-ClyF was immobilized on the surface of silicone catheter for 2 h at room temperature, inoculated with ~10^6^ CFU/mL of *S. aureus* N315 for 3 h at room temperature, and then fabricated into a flowing peripherally inserted central catheter with a fluid speed of 0.6 mL/min (Figure 6a). Staphylococcal biofilms formed on the surface of silicone catheter after 24 h of media flow was determined by plating assay. Results showed that a significant drop (*p* < 0.001) in bacterial burden in the SiBP1-ClyF immobilized silicone catheters, indicating an average bacterial load of 2.2-log10 cfu/cm^2^, compared to that of the mock-immobilized PBS-treated control catheters (5.8-log10 cfu/cm^2^; Figure 6b). The decreased bacterial load can be correlated with the high staphylolytic activity of SiBP1-ClyF on surfaces of silicone catheters in the continuous flow model.

### 2.6. SiBP1-ClyF Immobilized Surface Supports Normal Growth of Mammalian Cells

**Figure 7:**
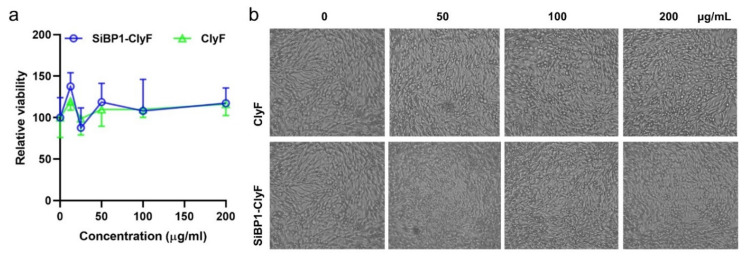
SiBP1-ClyF-immobilized surfaces support normal growth of mammalian cells. Wells were immobilized with various concentrations (0, 12.5, 25, 50, 100, and 200 μg/mL) of SiBP1-ClyF or ClyF overnight at 4 °C and inoculated with 104 cells/well of BHK-21 cells for 24 h, the cell viability of each treatment was then determined by CCK-8 assay (**a**) and the cell confluence of each treatment was captured by microscopy (**b**). Representative images under 10× objective lens from wells immobilized with 0, 50, 100, and 200 μg/mL SiBP1-ClyF or ClyF were shown.

To understand the potency of SiBP1-ClyF-functionalized surface in clinic applications, we evaluated the cytotoxicity of those surfaces by CCK-8 assay. To this end, BHK-21 cells were inoculated in wells immobilized with various concentrations of SiBP1-ClyF for 24 h, the cell viability was then determined. Results showed that rare differences in cell viability were observed from SiBP1-ClyF-immobilized wells and PBS-treated control wells (Figure 7a), suggesting that SiBP1-ClyF-functionalized surface could support the normal growth of mammalian cells. Note that similar biocompatibility was also observed in ClyF-immobilized wells (Figure 7a), indicating the safety profile of native ClyF, which is also consistent with our previous in vivo observations [14]. As expected, SiBP1-ClyF and ClyF immobilized wells supported a normal cell proliferation, with similar confluence to that of the mock-immobilized control wells (Figure 7b).

## 3. Discussion

In recent decades, antibiotic resistance has emerged as a mounting menace for global healthcare [35]. Potential pathogens have established themselves as multidrug-resistant superbugs, which are refractory to conventional antibiotics. With scarce treatment options, many of the untreatable bacterial infections are now primarily cause of mortality globally. The current pipeline for new antibiotics is drying, which is even more vexatious for healthcare professionals. Currently, clinicians and researchers are forced to look for alternative agents to antibiotics, including bacteriophages and their lysins, antibacterial metal nanoparticles, phytochemicals, antimicrobial peptides, nitric oxide, and other secondary metabolites [36,37,38,39,40,41]. Lysins have gained much popularity because of their high bactericidal properties, rapid killing activity, low risk of resistance, and modular proteinaceous structure. However, the performance of lysins on solid surfaces as a means to address DRIs is still largely unknown. In this study, we evaluated the bactericidal and antibiofilm activity of the chimeric staphylolytic lysin ClyF on various clinic relevant surfaces by genetically engineering with SiBPs and found that lysin-functionalized surfaces gain improved resistant to bacterial adhesion and biofilm formation.

Immobilization of enzymes may reduce their catalytic activities because of the steric restriction and limited access to the substrate to enzymatic site in particular conformation [42]. Therefore, in the present study, ClyF was designed to modulate with different peptides to optimize a suitable one that can help ClyF to conquer steric restriction and conformational hindrance for immobilized bacteriolytic activity. By adopting flexible linkers, peptides could thus take any orientation, including a folding back on the lysin structure. The linker can contribute to an increased autonomy of each moiety, i.e., ClyF for killing and peptide for silica binding. In this regard, two ClyF variants, especially SiBP1-ClyF, exhibited satisfactory properties for immobilization applications, as evidenced by their high antibacterial and antibiofilm capacities against *S. aureus* planktonic cells and biofilms on different surfaces. 

Notably, one SiBP-construct, i.e., SiBP3-ClyF showed impaired bactericidal activity. Increase of activity of lysins acting against Gram-positive bacteria by positively charged peptides has been documented previously [43,44]; therefore, the positive charges present in SiBP1 (Z = +1) and SiBP2 (Z = +6) may explain their enhanced activities against planktonic strains. It may thus be the lack of a positive charge and the additional steric hindrance of the fused peptide SiBP3 (Z = 0) that lowers the activity of SiBP3-ClyF, although the detailed mechanisms behind still need further study. However, for the immobilized enzymes, the SiBP is involved in binding the silica-based surfaces, thus would then not be further available to interact with the negatively charged bacterial cell wall. Nonetheless, the influence of the charge of silica-based materials on the observed activity of SiBP-fusions still needs to be established.

A potential shortcoming of the current approach is the limitations of the recycling potential and long-term stability of ClyF-immobilized surfaces. This may be due to the fragility of biological protein molecules which tend to lose their activity when in contact with a support surface [45]. According to the current progress in immobilizing functional enzymes on solid supports, several approaches could be adopted to solve such problem, for instance, optimizing the storing buffer of coated surfaces [26], favoring the molecular orientation of immobilized enzymes through rational design [46], fabricating ClyF to bioactive films by conjugating with nanoparticles [47], self-assembling spider silk protein [48], or programable biofilm-integrated nanofiber [49]. 

Biofilms are complex 3D structure of bacterial communities, which provides enhanced bacterial colonization on solid surfaces and extravagant the management of infections [50]. Biofilms provide protection to bacteria from host immunity and block the access of antibiotics by producing thick extracellular polymeric substances [51,52]. Medical DRIs are biologically initiated from microorganisms’ adherence on surface of interaction. Therefore, preventing bacterial attachment and growth via bactericidal lysin-mediated surface immobilization is a fundamental and efficient strategy to prevent DRIs. Taking *S. aureus* as an example, the present study showed that immobilization staphylolytic lysin ClyF via silica-targeting peptide SiBP1, i.e., SiBP1-ClyF, on surfaces can not only lead to scarce bacterial colonization and growth due to lysis of attached bacteria, but also resulted in significantly reduced mature biofilms developed under both static and dynamic conditions. However, the influence of lysed bacterial debris on the ecosystem in the long run still needs further study.

Because of the nature of phage, lysins are usually specific to their target bacterium, commonly in genus level, which makes them different from broad-spectrum antibiotics. As a proof-of-concept study, we take staphylococci-targeted lysin, ClyF, as an example to show the feasibility of using lysin-functionalized surfaces as a new approach to prevent DRIs. Our current model, in principle, will only be active against bacterial species that are susceptible to the killing of ClyF, but not other strains that lie outside of the lysis spectrum of ClyF. Other bacteria could also form biofilms; our current strategy, however, could be transplanted to another bacterium by using an ideal lysin targeting that bacterium, considering the continuous progress in lysin discovery and engineering. Therefore, the present strategy provides a roadmap for endolysin immobilization to counter device-related drug-resistant infections, which are cumbersome to conventional antibiotic therapy. 

## 4. Materials and Methods

### 4.1. Construction of SiBP-ClyF Fusions

The ClyF-coding gene was amplified with three different SiBPs by overlap polymerase chain reaction assays with specific primers (Table S4). The amplified SiBP-ClyF gene products were cloned into *Nco*I and *Xho*I sites of pET28b(+) vector and further transformed into *E. coli* BL21(DE3) competent cells. Clones harboring the appropriate sequence were confirmed by sequencing (Table S4) and then processed for further experiments.

### 4.2. Protein Expression and Purification

A single positive colony for ClyF and its SiBP-ClyF constructs was cultured separately in 5 mL of lysogeny broth (LB) supplemented with 50 μg/mL kanamycin. The tubes were incubated at 220 rpm at 37 °C overnight. The next day, 1 mL of the culture was transferred to 100 mL of fresh LB medium with 50 μg/mL kanamycin and incubated with shaking at 37 °C for 2–3 h. Protein expression was induced with 0.25 mM isopropyl-β-D-thiogalactoside when the optical density (600 nm) of inoculated media reached 0.6–0.8. The flasks were incubated for 16–20 h at 16 °C with 120 rpm of shaking. Cells were harvested by centrifugation at 12,000 rpm at 4 °C for 20 min and cell pellet was washed thrice with Tris-HCl buffer (50 mM sodium phosphate, 500 mM NaCl, pH 8) to remove media components. The cells were lysed using pressure facture and purified by Ni-NTA affinity chromatography with gradient of imidazole solutions. Purified enzyme was dialyzed against 0.1 mM CaCl_2_ containing phosphate-buffered saline (PBS, pH 7.4) to remove imidazole and confirmed by SDS-PAGE analysis [53]. The dialyzed protein was then filter sterilized using 0.22 μm syringe filters and stored at 4 °C for subsequent experiments. 

### 4.3. Structure Prediction of ClyF and Its Variants

The 3D structure of ClyF and its variants were predicated by RoseTTAFold online service [53] and further analyzed by PyMOL.

### 4.4. Nano Differential Scanning Fluorimetry

The thermal stability of ClyF and its variants were analysed by a nano differential scanning fluorimetry method using a Prometheus NT.48 instrument (NanoTemper Technologies, San Francisco, CA, USA). The intrinsic emission fluorescence of each protein (200 μg/mL) at 350 and 330 nm was monitored over a temperature range of 25 to 90 °C (increasing step of 1 °C/min), using dialysis buffer as controls. The first derivative of the fluorescence ratio at 350 nm and 330 nm (1st derivative of F350/F330) was calculated automatically by the PR-ThermControl software supplied with the instrumentation. Samples were measured in triplicates. The thermal unfolding transition temperature (Tm) corresponds to peaks of the 1st derivative of F350/F330.

### 4.5. Circular Dichroism

The circular dichroism spectra of ClyF and its variants (200 µg/mL) were collected with an Applied Photophysics Chirascan Plus circular dichroism spectrometer (Leatherhead, UK) from 190–260 nm (0.1 cm path length) at room temperature. The spectra of air and buffer were recorded as background and baseline, respectively. The secondary structure was analyzed by the CDNN V2.1 software, supplied by the instrument manufacturer.

### 4.6. Bactericidal Activity of Free ClyF and Its SiBP-Fused Variants

Antibacterial activity of ClyF and its SiBP-fused variants were evaluated by log killing assay as described previously [14]. Briefly, *S. aureus* strains (Table S3) were inoculated in 5 mL of LB broth at 37 °C. Next day, 50 µL of overnight grown culture was inoculated to 5 mL of fresh LB media for 3–4 h at 37 °C with shaking. Cells were then harvested, washed, and resuspended in PBS to an optical density of OD_600_ = 0.6. Bacterial suspension was treated with an equal molar concentration (final concentration of 0.7 μM, corresponding to ClyF concentration of 20 μg/mL) of ClyF and its SiBP-fused variants for 1 h at 37 °C. The turbidity of each treatment was monitored by a Synergy H1 microplate reader and viable bacterial quantification was determined by plating serial 10-fold PBS-diluted dilutions on LB agar. Parallel suspensions that treated with an equal volume of PBS were used as controls.

### 4.7. Antibacterial Activity of Lysin-Functionalized Surfaces

To evaluate the bactericidal activity of lysin-functionalized silicon glass, ClyF and its SiBP-fused variants were immobilized in glass 96-well plates at an equal molar concentration of 1.75 μM (corresponding to ClyF concentration of 50 μg/mL) for 2 h at room temperature. Wells were then washed twice with PBS, inoculated with ~10^6^ CFU/well of *S. aureus* N315 in LB medium, and incubated at 37 °C. The growth of bacteria was determined by monitoring the OD_600_ in each well using a microplate reader for 20 h at intervals of 15 min. Additionally, the viable bacterial number in each well after incubation for 1 h was further confirmed by plating serial dilutions on LB agar. The capacity of immobilized lysin in each treatment was expressed as the percentage of the reduction in bacterial cell count compared to that of the PBS-treated groups (%log reduction).

To evaluate the performance of ClyF variants on different surfaces, siliconized glass coverslip, silicone-coated latex catheter (φ = 6.7 mm, ~1 cm length; 20Fr, STAR, Zhanjiang, China), and silicone catheter (φ = 6.7 mm, ~1 cm length; 20Fr, STAR, Zhanjiang, China) were coated with different concentrations (0, 20, 40, 60, 80, and 100 μg/mL) of ClyF, SiBP1-ClyF, or SiBP2-ClyF for 2 h at room temperature in 48-well plates. These surfaces were washed twice with PBS and then inoculated with ~10^6^ CFU/well of PBS-suspended *S. aureus* N315 for 1 h at 37 °C. Viable bacterial number was quantified by plating serial dilutions on LB agar. Each experiment was carried out in biological triplicates. 

### 4.8. Recycle Capacity and Stability of Lysin-Functionalized Surface

To evaluate the recycle capacity of lysin-functionalized silicon glass, ClyF and its SiBP-fused variants were immobilized in glass 96-well plates at an equal molar concentration of 3.5 μM (corresponding to ClyF concentration of 100 μg/mL) for 2 h at room temperature. Wells were then washed twice with PBS, inoculated repeatedly with ~106 CFU/well of *S. aureus* N315 and incubated at 37 °C for 10 min for 5 cycles. Viable bacterial number from each cycle was further confirmed by plating serial dilutions on LB agar. The relative capacity of each enzyme was compared to the capacity of SiBP1-ClyF (%log reduction) in the first cycle. 

To ascertain the stability of lysin-functionalized surface during storage, glass 96-well plates coated with an equal molar concentration of 1.75 μM (corresponding to ClyF concentration of 50 μg/mL) ClyF and its SiBP-fused variants were stored for different times at 4 °C. The relative capacity of lysin-functionalized wells was determined as described above at 0, 1, 2, 3, 4, 5, 6, 7, and 10 days post-immobilization, and normalized to the bactericidal activity of corresponding wells before storage. All experiments were carried out in biological triplicates. 

### 4.9. Prevention of Biofilm Formation on Lysin-Functionalized Surfaces

Siliconized glass coverslips were coated with 100 µg/mL of SiBP1-ClyF for 2 h at room temperature in 6-well plates, washed twice with PBS, and then incubated with ~106 CFU/well of *S. aureus* N315 in 2 mL TBSG (1.5% tryptone, 0.5% soytone, 0.5% NaCl, and 1% glucose) at 37 °C. Biofilms developed at 2, 6, 10, and 24 h were analyzed by a JSM-6390 scanning electron microscope (JEOL, Tendo, Japan) as described previously [54]. Groups coated with ClyF and PBS were used as controls. Additionally, biofilms established for 10 h were further stained with Live/Dead Baclight bacterial viability kit (L13152, Thermo, Shanghai, China) and visualized by an UltraVIEW VoX confocal fluorescence microscope (PerkinElmer, Waltham, MA, USA) as described previously [55]. PBS-treated mock-coated groups were used as controls. 

To test the capacity of lysin-coated surface against flow biofilms, silicone catheter (φ = 4 mm, ~4 cm length; 20Fr, STAR, Zhanjiang, China) was coated with 100 µg/mL of SiBP1-ClyF for 2 h at room temperature. The coated catheters were washed twice with PBS and then incubated with ~10^6^ CFU/mL of *S. aureus* N315 for 3 h at room temperature to allow bacterial attachment on the catheter surface. Afterwards, the *S. aureus*-containing silicone catheter was inserted into a peripherally inserted central catheter and flowed with LB at a fluid speed of 0.6 mL/min for 24 h to allow the biofilm establishment. Finally, silicone catheters were removed and underwent an ultrasound bath for 10 min to disrupt established biofilms. Resulted viable bacterial number was analyzed by plating serial dilutions on LB agar. PBS-treated mock-coated silicone catheter was used as control.

### 4.10. Cytotoxicity of Lysin-Functionalized Surface

Various concentrations of ClyF and SiBP1-ClyF (0, 12.5, 25, 50, 100, and 200 μg/mL) were immobilized in 96-well plates overnight at 4 °C. Wells were then washed twice with PBS and inoculated with 10^4^ cells/well of BHK-21 cells in Dulbecco’s modified Eagle’s medium (DMEM; Sigma-Aldrich, Shanghai, China) supplemented with 10% fetal bovine serum, 1% penicillin, and 1% streptomycin in a humidified atmosphere of 5% CO_2_ at 37 °C for 24 h. Afterwards, the cell viability of each treatment was determined by CCK-8 assay and the cell confluence was captured by microscopy. For CCK-8 assay, the contents of the plates were replaced with fresh medium containing 10% CCK-8 solution and incubated at 37 °C for 1 h. The final optical density at OD_450_ was noted by a Synergy H1 microplate reader (BioTek, Winooski, VT, USA). The results were expressed as relative cell viability, expressed as a percentage of the growth of cells in control wells immobilized with PBS only. For confluence detection, wells were imaged by an IX51 inverted microscope under 10× objective lens (Olympus, Center Valley, PA, USA). All experiments were carried out in biological triplicates.

### 4.11. Statistic Analysis

Data analyses were performed by one-way analysis of variance (ANOVA) and layout by GraphPad Prism 8.0.

## 5. Conclusions

The present study demonstrates the antibacterial and antibiofilm potential of immobilized ClyF on multiple solid surfaces via silica-binding peptides. One such variant, SiBP1-ClyF, showed not only feasible immobilization stability on solid support surfaces, but also retains high antibacterial and antibiofilm abilities. This study supports a promising approach to design innovative antimicrobial surfaces by using bactericidal lysins, which can be highly selective for their target bacteria. The generic approach reported here could also be easily extended to other pathogen-targeted lysins to prevent multiple medical device-related drug-resistant infections.

## Data Availability

The data presented in this study are available from the corresponding author upon request.

## Supplementary Materials

The following are available online at https://www.mdpi.com/article/10.3390/ijms222212544/s1.
