# Supplementary material for: Optimized Silica-Binding Peptide-Mediated Delivery of Bactericidal Lysin Efficiently Prevents Staphylococcus aureus from Adhering to Device Surfaces
Source: Int J Mol Sci. 2021 Nov 21;22(22):12544. doi: 10.3390/ijms222212544 (PMC8619460; doi:10.3390/ijms222212544)

Supplementary materials

Optimized silica-binding peptide-mediated delivery of bactericidal lysin efficiently prevents *Staphylococcus aureus* from adhering to device surfaces

Wan Yang[1], Vijay Singh Gondil[2], Dehua Luo[1], Hongping Wei[2,3], Jin He[1], Hang Yang[2,3]*

[1]State Key Laboratory of Agricultural Microbiology, College of Life Science and Technology, Huazhong Agricultural University, Wuhan 430070, China;

[2]CAS Key Laboratory of Special Pathogens and Biosafety, Center for Biosafety Mega-Science, Wuhan Institute of Virology, Chinese Academy of Sciences, Wuhan, Hubei, 430071, China.

[3]University of Chinese Academy of Sciences, Beijing 100049, China.

For correspondence: Hang Yang: E-mail: yangh@wh.iov.cn; Tel.: (+86) 27 51861078; Fax: (+86) 27 87199492.

Table S1. Secondary structure of ClyF variants.

Secondary Structure	Percentage of composition (%)			
	ClyF	SiBP1-ClyF	SiBP2-ClyF	SiBP3-ClyF
Helix	16.4	16.8	15.9	16.5
Antiparallel	26.1	25.3	27.4	26.1
Parallel	15.4	15.2	15.7	15.3
Beta-Turn	20.7	20.6	20.9	20.7
Rndm. Coil	47.3	46.9	47.5	46.9

Table S2. Biochemical properties of ClyF variants.

Protein	p*I*	Mw (Da)	Tm (°C)
ClyF	9.57	29416.83	52.1 ± 0.13
SiBP1-ClyF	9.63	31572.12	52.9 ± 0.30
SiBP2-ClyF	9.8	32114.77	54.3 ± 0.14
SiBP3-ClyF	9.57	31494.07	51.6 ± 0.062

Table S3. Strains used in this study.

Organism	Strain	Susceptibility[c]	SCCmec	Source[d]
S. aureus	WHS11103[a]	Ox^R	NA	1
	WHS11032[a]	Ge^R Ox^R	IV	1
	WHS11005[a]	Ge^R Ox^R	I	1
	WHS11018	Ge^R Ox^R	II	1
	WHS11011[a]	Ge^R Ox^R	II	1
	CCTCC AB91118[b]	Ox^S	NA	1
	RN4220[b]	Ox^S	NA	1
	N315[a]	Ge^R Ox^R	II	1

[a]: Methicillin-resistant *Staphylococcus aureus*; [b]: Methicillin-sensitive *Staphylococcus aureus*;

[c]Susceptibility: Ox: oxacillin; Ge: gentamycin; R: resistant; S: sensitive;

[d]Source: 1. Laboratory collection.

Table S4. Primers, silica-binding peptides and proteins used in this study.

Name	Sequence (5'-3')
**Primers**	
SiBP-ClyF-F	tataccatggcactgcctaaaacgggtaaac
SiBP1-ClyF-R1	gcgggctcatgccgctgcccgccgcgctgcccgcgctgccaaatgtaaccccaagcattgc
SiBP1-ClyF-R2	ggaggatcctccggtatgatgatggcgcggatgcggatgcgggctcaatgccgctgccc
SiBP1-ClyF-R3	atatctcgagggatccggaggatcctccggtatg
SiBP2-ClyF-R1	ttttgctgctgccgctgcccgccgcgctgcccgcgctgccaaatgtaccccaagcattgc
SiBP2-ClyF-R2	cggcgtttgctgcctttgctgccgctatagctgccgctttttttgctgctgccgctgccc
SiBP2-ClyF-R3	atatctcgagggatccggaggatcctcccagaatgcggcgtttgctgcctttgct
SiBP3-ClyF-R1	tcggcggatggccgctgcccgccgcgctgcccgcgctgccaaatgtaccccaagcattgc
SiBP3-ClyF-R2	Ccggaggatcctccatgcatatgcggatggctcgcgttcatcggcggatggccgctgccc
SiBP3-ClyF-R3	atatctcgagggatccggaggatcctccatgc
**Peptides/protein**	
SiBP1	MSPHPHPRHHHT
SiBP2	SSKKSGSYSGSKGSKRRIL
SiBP3	HPPMNASHPHMH

ClyF	MALPKTGKPTAKQVVDWAINLIGSGVDVDGYYGRQCWDLPNY<br>IFNRYWNFKTPGNARDMAWYRYPEGFKVFRNTSDFVPKPGDIA<br>VWTGGNYNWNTWGHTGIVVGPSTKSYFYSVDQNWNNSNSYV<br>GSPAAKIKHSYFGVTHFVRPAYKAEPKPTPPGTPPGTVAQSAPNL<br>AGSRSYRETGTMTVTVDALNVRRAPNTSGEIVAVYKRGESFDY<br>DTVIIDVNGYVWVSYIGGSGKRNYVATGATKDGKRFGNAWGTF<br>K
SiBP1-ClyF	MALPKTGKPTAKQVVDWAINLIGSGVDVDGYYGRQCWDLPNY<br>IFNRYWNFKTPGNARDMAWYRYPEGFKVFRNTSDFVPKPGDIA<br>VWTGGNYNWNTWGHTGIVVGPSTKSYFYSVDQNWNNSNSYV<br>GSPAAKIKHSYFGVTHFVRPAYKAEPKPTPPGTPPGTVAQSAPNL<br>AGSRSYRETGTMTVTVDALNVRRAPNTSGEIVAVYKRGESFDY<br>DTVIIDVNGYVWVSYIGGSGKRNYVATGATKDGKRFGNAWGTF<br>K GSAGSAAGSG MSPHPHPRHHHT
SiBP2-ClyF	MALPKTGKPTAKQVVDWAINLIGSGVDVDGYYGRQCWDLPNY<br>IFNRYWNFKTPGNARDMAWYRYPEGFKVFRNTSDFVPKPGDIA<br>VWTGGNYNWNTWGHTGIVVGPSTKSYFYSVDQNWNNSNSYV<br>GSPAAKIKHSYFGVTHFVRPAYKAEPKPTPPGTPPGTVAQSAPNL<br>AGSRSYRETGTMTVTVDALNVRRAPNTSGEIVAVYKRGESFDY<br>DTVIIDVNGYVWVSYIGGSGKRNYVATGATKDGKRFGNAWGTF<br>K GSAGSAAGSG SSKKSGSYSGSKGSKRRIL

SiBP3-ClyF	MALPKTGKPTAKQVVDWAINLIGSGVDVDGYYGRQCWDLPNY<br>IFNRYWNFKTPGNARDMAWYRYPEGFKVFRNTSDFVPKPGDIA<br>VWTGGNYNWNTWGHTGIVVGPSTKSYFYSVDQNWNNSNSYV<br>GSPAAKIKHSYFGVTHFVRPAYKAEPKPTPPGTPPGTVAQSAPNL<br>AGSRSYRETGTMTVTVDALNVRRAPNTSGEIVAVYKRGESFDY<br>DTVIIDVNGYVWVSYIGGSGKRNYVATGATKDGKRFGNAWGTF<br>K GSAGSAAGSG HPPMNASHPHMH

Figure S1. Image showing the molecular weight and purity of ClyF variants. Lanes 1, 2, 3, and 4 show standard molecular ladder (M), SiBP1-ClyF, SiBP2-ClyF, SiBP3-ClyF, and ClyF, respectively.

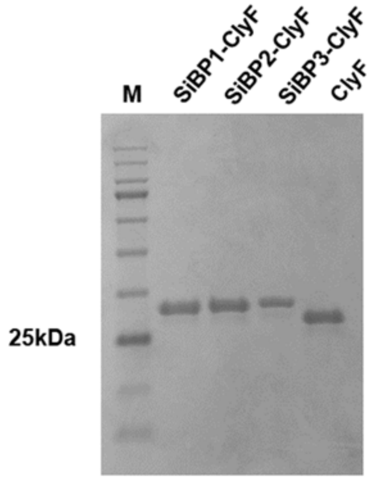

Figure S2. Predicted structure of ClyF and its SiBP variants by RoseTTAFold. The catalytic domain of four proteins were labelled in blue, cell-wall binding domain in green, and SiBP in red. The confidence of prediction for ClyF, SiBP1-ClyF, SiBP2-ClyF, SiBP3-ClyF was 0.79, 0.73, 0.71, and 0.74, respectively.

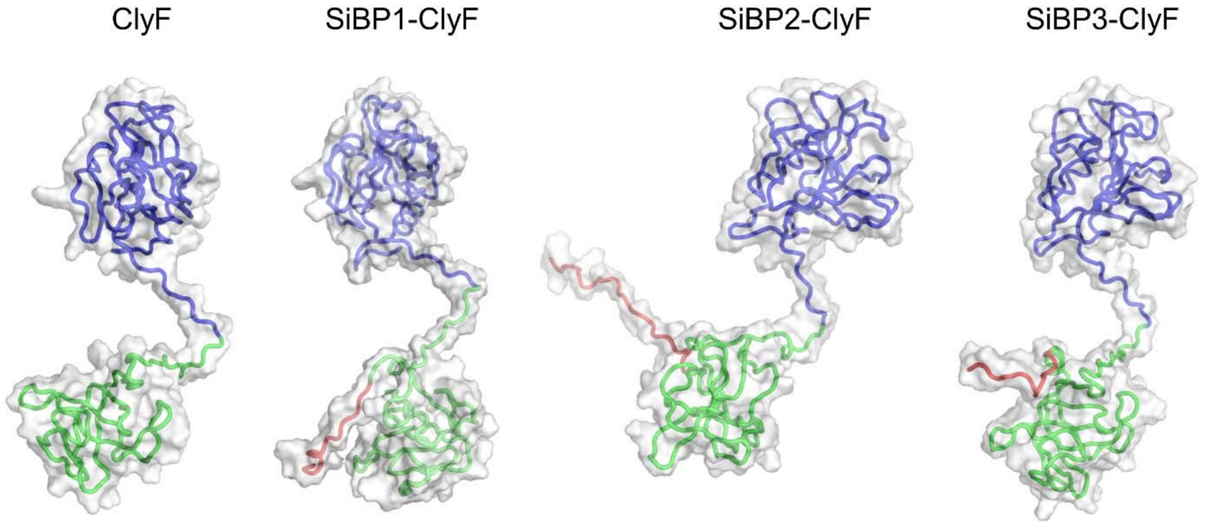

Figure S3. Bacteriolytic activity of ClyF and its variants. Eight *S. aureus* strains were treated with an equal molar concentration of 0.7 μM ClyF or its variants for 1 h at 37°C. The turbidity of each treatment was monitored by a Synergy H1 microplate reader.

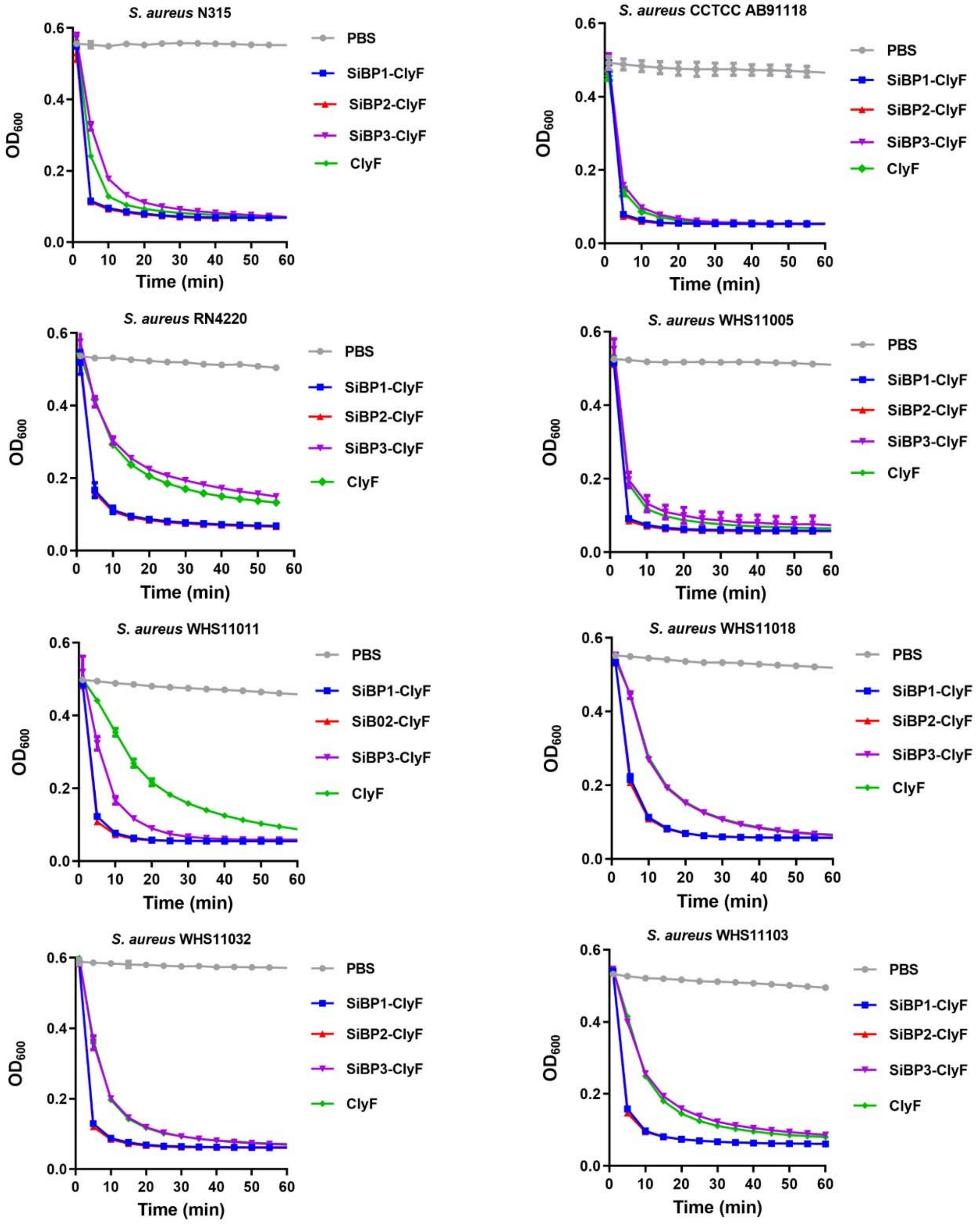

Supplement: Supplementary file 1 [file ijms-22-12544-s001.zip › ijms-1446964-supplementary.pdf]
